# Grouting Quality Evaluation in Post-Tensioning Tendon Ducts Using Wavelet Packet Transform and Bayes Classifier

**DOI:** 10.3390/s19245372

**Published:** 2019-12-05

**Authors:** Xiang-Tao Sun, Dan Li, Wen-Yu He, Zuo-Cai Wang, Wei-Xin Ren

**Affiliations:** School of Civil Engineering, Hefei University of Technology, Hefei 230009, China; sunxt@mail.hfut.edu.cn (X.-T.S.); wyhe@hfut.edu.cn (W.-Y.H.); wangzuocai@hfut.edu.cn (Z.-C.W.); renwx@hfut.edu.cn (W.-X.R.)

**Keywords:** tendon ducts, grouting defects, wavelet packet transform, Bayes classifier, piezoelectric transducers

## Abstract

The grouting quality of tendon ducts is very important for post-tensioning technology in order to protect the prestressing reinforcement from environmental corrosion and to make a smooth stress distribution. Unfortunately, various grouting defects occur in practice, and there is no efficient method to evaluate grouting compactness yet. In this study, a method based on wavelet packet transform (WPT) and Bayes classifier was proposed to evaluate grouting conditions using stress waves generated and received by piezoelectric transducers. Six typical grouting conditions with both partial grouting and cavity defects of different dimensions were experimentally investigated. The WPT was applied to explore the energy of received stress waves at multi-scales. After that, the Bayes classifier was employed to identify the grouting conditions, by taking the traditionally used total energy and the proposed energy vector of WPT components as input, respectively. The experimental results demonstrated that the Bayes classifier input with the energy vector could identify different grouting conditions more accurately. The proposed method has the potential to be applied at key spots of post-tensioning tendon ducts in practice.

## 1. Introduction

Post-tensioning technology is widely used in bridge construction, which can reinforce concrete beams and increase their bearing capacity. Filling the tendon duct with grouting mortar is an important procedure of this technology in order to protect the prestressing reinforcement from environmental corrosion and to make a smooth stress distribution. Unfortunately, various grouting defects including cavities, partial grouting, and even no grouting may occur inside the tendon ducts. The prestressing reinforcement would seriously corrode due to water, air, and chlorides when defects exist in the duct. The corrosion of the reinforcement could further lead to bridge collapse without forewarning [1,2]. It is difficult to assess the grouting condition of tendon ducts in post-tensioning structures because they are cast inside the concrete. Therefore, there is an urgent need for efficient methodologies to evaluate the grouting quality of tendon ducts.

Many non-destructive testing (NDT) techniques, such as ultrasonic echo, impact echo, ultrasonic tomography, and ground penetrating radar, have been applied to detect grouting defects in post-tensioning tendon ducts. Krause et al. [3] investigated the grouting quality of tendon ducts using ultrasonic echo methods. The propagation of elastic waves in the test specimen was modeled by the elastodynamic finite integration technique. Lu et al. [4] explored the effect of materials, the shape of prestressing tendon duct, and the wave velocity difference between concrete and grouting mortar on ultrasonic tomography for evaluating grouting quality. The ultrasonic tomography technique was suitable for evaluating the grouting quality when the wave velocity difference was small. However, its accuracy needs to be further studied. Muldoon et al. [5] tested a series of concrete beams with tendon ducts using impact echo and concluded that the impact echo technique cannot detect grouting defects in plastic ducts. Olson et al. discussed the feasibility and efficiency of impact echo scanning (IES) technique in grouting quality evaluation [6]. Li et al. [7] applied the Hilbert-Huang transformation (HHT) to analyze impact echo signals to assess the grouting compactness in tendon ducts, and reported that the thickness frequencies decreased gradually with the decrease of grouting compactness. Giannopoulos et al. [8] detected voids in post-tensioning concrete beams by ground penetrating radar. However, the steel bars inside the structures would cause strong interference to electromagnetic wave and this technique was found unsuitable for metal ducts. Terzioglu et al. [9] compared the performance of several NDT techniques in detecting the location and severity of fabricated grouting defects in a full-scale post-tensioning specimen. Even though both impact echo and ultrasonic echo techniques were effective in identifying the location of grout defects, they could not differentiate between water, void, or compromised grout conditions. Overall, more researches are needed for NDT techniques that are applicable to complex anchorage and multiple-duct regions and that are able to evaluate the severity and nature of grouting defects.

In recent years, piezoelectric materials have been widely applied in ultrasonic-, stress wave-, and electromechanical impedance-based structural defect detection because of their wide bandwidth and low price [10,11,12,13]. Kawiecki et al. [14] verified the feasibility of damage detection by active sensing approach using a number of piezoelectric patches placed on the surface of a concrete block. Michaels [15] used piezoceramic patches as distributed ultrasonic sensors to detect, locate, and characterize structural damage in aluminum plates. Ihn et al. [16] investigated a diagnostic technique for monitoring crack growth in metallic structures using built-in piezoelectric transducers. Wu et al. [17] combined piezoelectric transducers and fiber Bragg grating (FBG) to form a hybrid distributed sensor network in order to detect the damage in composite plates. The distinguished advantage of this hybrid detection system was that the sensors would not interfere with each other because of different working principles. Zima et al. [18] used piezoelectric ceramic patches to generate ultrasonic guided wave for identifying debonding between bars and grouting mortar in ground anchors. Lim et al. [19,20] developed a semi-analytical model of surface bonded piezoelectric based wave propagation technique for concrete strength monitoring during the curing process. Malinowski et al. [21] applied piezoelectric sensors in the electromechanical impedance-based method to detect weak adhesive bonds of carbon fiber reinforced polymer. Kudela et al. [22] applied the piezoelectric array to crack detection in an aluminum plate and proposed a novel strategy based on Lamb waves, which helped to increase the damage imaging resolution and crack detection accuracy. Lead zirconate titanate (PZT) is the most commonly used piezoelectric material because PZT has strong piezoelectricity, high dielectric constants, and availability of different shapes. Chiu et al. [23] utilized piezoceramic transducers for the crack damage detection of full-size shear-critical high-strength reinforced concrete (HSRC) column members. However, the application of PZT was limited in civil engineering due to its fragility. Song et al. [24,25] thus proposed the concept of smart aggregate (SA), i.e., PZT patch with marble protection, which can be easily embedded inside concrete structures and effectively identify the existence and severity of cracks. Feng et al. [26] successfully monitored the grouting progress of concrete-filled steel tubes through an active sensing setup with combined SAs and piezoceramic patches and a wavelet packet-based energy index matrix of received signals. Up to date, various SAs have been investigated in the health monitoring of civil engineering structures [27,28,29,30,31].

There are few researches on the grouting compactness monitoring in post-tensioning tendon ducts using PZT transducers. Jiang et al. applied piezoelectric transducers to monitor grouting compactness in a post-tensioning tendon duct on a cross section [32]. One transducer bonding on the prestressing reinforcement was used as actuator. Two PZT patches mounting on the surface of the tendon duct were used as sensors. Experimental results indicated that the proposed method had the potential to monitor the grouting compactness on a cross section in real time. Then, Jiang et al. conducted the finite element simulation of wave prorogation in a tendon duct [33]. Tian et al. applied the PZT ring actuator to adapt the prestressing steel bar better [34]. However, these researches were concerned on monitoring the grouting level in a cross section and did not consider other typical grouting defects in practice.

The ultrasonic stress waves received by the PZT sensors are non-stationary signals, whose power spectra change with time. Time-frequency analysis, such as wavelet transform (WT) and wavelet packet transform (WPT), is able to reveal their frequency components more accurately than frequency analysis like fast Fourier transform (FFT) [35]. It also helps to suppress the influence of noise whose energy is generally in low frequency scales [36]. The frequency components of ultrasonic stress waves generally have diverse sensitivities to various structural defects, and can be used to identify defects more precisely than the waveforms in the time domain [37]. Moreover, indexes such as energy and entropy are established to quantify the wavelet coefficients or reconstructed frequency components before identification. If any defect exists along the propagation path of ultrasonic waves, the energy of the signals received by the sensors would reduce significantly. The energy of WPT has therefore been widely used in defect detection. Yu et al. [38] employed the energy vector of WPT components of ultrasonic signals for damage detection in wood utility poles. Jiang et al. [32] used the energy of WPT components of stress waves received by PZT transducers in order to classify different grouting conditions. Yang et al. [39] applied the energy of WPT components of stress waves received by embedded PZT transducers to evaluate the soil compaction.

In this study, the detection of various grouting defects in post-tensioning tendon ducts, including partial grouting and cavity defects, was studied using stress waves generated and received by piezoelectric transducers. A method based on WPT and Bayes classifier was proposed to evaluate the grouting quality. Six typical grouting conditions with both partial grouting and cavity defects of different dimensions were experimentally investigated. The energy of received signals attenuated to varying degrees in different grouting conditions. The patterns involved in the energy distribution of received signals at multi-scales were investigated and utilized to identify different grouting conditions. The Bayes classifier was applied to take into account the randomness of practical measurements. Finally, the energy vector of WPT components was proposed as the input of Bayes classifier, and was proved to perform better than the traditionally used total energy in the classification of different grouting conditions.

## 2. Methodology

**Figure 1:**
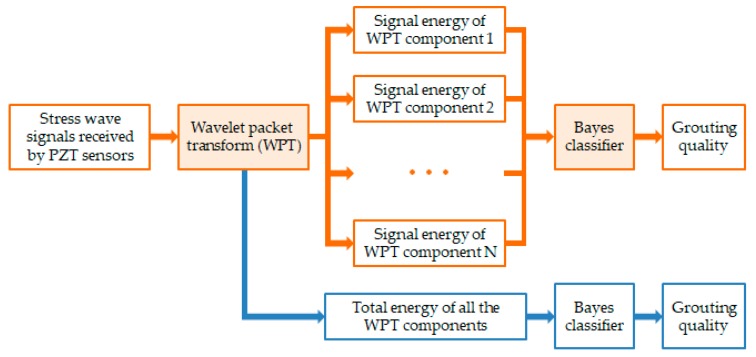
Overview of the proposed grouting quality evaluation method.

The grouting quality evaluation method proposed in this study was based on WPT and Bayes classifier. As the stress wave propagated in the grouted tendon duct, it would attenuate to different degrees depending on the grouting quality. The WPT was therefore applied to explore the energy distribution of received signals at multi-scales. Considering that the energy of stress wave received in different grouting qualities may overlap and that the grouting conditions could be very complex in practice, a Bayes classifier was further employed to identify the types and dimensions of grouting defects by taking the energy vector of WPT components as the input. The schematic of the proposed method is presented in Figure 1. For the comparison, the traditionally used total energy of WPT components was also input to another Bayes classifier for evaluating the grouting quality.

### 2.1. WPT

WPT is a time-frequency analysis method developed based on WT. Compared with WT, its advantage is the complete level-by-level decomposition of nonstationary signals both in high-frequency regions and low-frequency regions [40]. WPT has been widely used to analyze non-stationary vibrational and ultrasonic signals in the field of structural health monitoring [25,31,41,42,43]. Here, WPT was introduced to decompose the received stress wave signals into different frequency scales and to explore the energies of different components.

Through j-level WPT, a voltage signal x(t) received by the PZT sensor could be decomposed into $2^{j}$ components, $x_{j}^{i}\left(t\right)$ ($i=1,2,\ldots ,2^{j}$).

(1) $$x\left(t\right)=\sum_{i=1}^{2^{j}}x_{j}^{i}\left(t\right)$$

The wavelet packet coefficients of the ith component $c_{j,k}^{i}$ is obtained by

(2) $$c_{j,k}^{i}=\int_{-\infty }^{\infty }x\left(t\right)\psi_{j,k}^{i}\left(t\right)dt$$

Here, $\psi_{j,k}^{i}$ are the wavelet packet functions that are orthogonal with each other. i, j, and k are the modulation, the scale, and the translation parameters, respectively. Each of the wavelet packet component $x_{j}^{i}\left(t\right)$ can be reconstructed by

(3) $$x_{j}^{i}\left(t\right)=\sum_{k=-\infty }^{\infty }c_{j,k}^{i}\psi_{j,k}^{i}\left(t\right)$$

**Figure 2:**
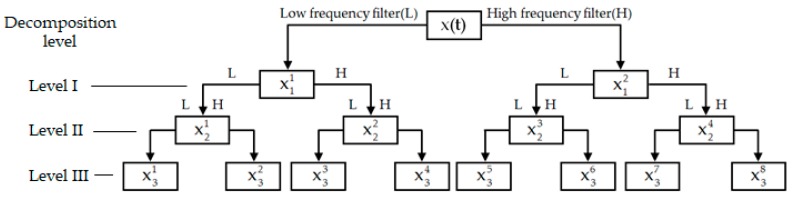
Procedure of the three-level wavelet packet transform (WPT).

Appropriate selection of the mother wavelet function and the decomposition level plays an important role in the performance of WPT on the analysis of practical signals. Among various mother wavelets, the family of Daubechies wavelets is orthogonal, compactly supported, and has good time-frequency localization property [44]. A Daubechies wavelet db2, which is widely used to analyze ultrasonic and vibration signals for structural damage detection [39,45,46], was applied as the mother wavelet function after trial and error in this study. A three-level WPT was determined to analyze the received signals after comparing the performance of different composition levels. As show in Figure 2, the original signal x(t) is decomposed into the approximation $x_{1}^{1}$ and the detail $x_{1}^{2}$ at the first composition level through the low- and high-frequency filters. At the second composition level, $x_{1}^{1}$ and $x_{1}^{2}$ are further decomposed into the approximations $x_{2}^{1}$ and $x_{2}^{3}$ as well as the details $x_{2}^{2}$ and $x_{2}^{4}$. The decomposition process is repeated as the composition level increased. After the three-level composition, the original signal is decomposed into eight frequency scales $x_{3}^{1}$, $x_{3}^{2}$, …, $x_{3}^{8}$. Obviously, WPT can achieve refined resolutions in both the low- and high-frequency regions through a series of quadrature mirror filters that are associated with the scaling function and the mother wavelet function [40]. It is thus able to reveal minor changes of analyzed signals in various frequency scales.

The signal energy of the ith WPT component $E_{i}$ is computed as

(4) $$E_{i}=\int_{-\infty }^{\infty }x_{j}^{i}{\left(t\right)}^{2}dt$$

The total energy of all the WPT components E is then calculated as

(5) $$E=\sum_{i=1}^{2^{j}}E_{i}$$

The total energy of all the WPT components was traditionally used as an index to distinguish structural defects of different types or dimensions. Six grouting conditions of different types or dimensions were experimentally investigated in this study. Theoretically, the total energy of WPT components for signals received in different grouting conditions increased strictly along with the increase of grouting compactness. However, it was found that the total energy of WPT components could have similar values for different grouting conditions and has certain randomness when a specimen with a specific grouting condition was tested multiple times. As a result, the value ranges of the total energy of WPT components for different grouting conditions overlapped with each other to various degrees, although their mean values were different. Misdiagnoses occurred if the total energy of WPT components was used solely as the classification criterion for grouting quality evaluation in practice. More details will be discussed in Section 4. Therefore, a machine learning method taking the respective signal energies of WPT components as an input vector was proposed to explore the energy distribution of received signals in the time-frequency domain and to classify the grouting conditions more accurately.

### 2.2. Bayes Classifier

Among various machine learning methods, Bayes classifier is a probabilistic classification model based on the Bayes theory. It has been proved to be a powerful tool for pattern recognition [47,48]. The mathematics of Bayes classifier used in this study is briefly introduced here. The general Bayes formula is given below

(6) $$P\left(A|B\right)=\frac{P\left(B|A\right)P\left(A\right)}{P\left(B\right)}$$

where P(A|B) is the probability of A under B condition. The Bayes classifier for grouting quality evaluation can be expressed as

(7) $$P\left(M_{n}|D,M\right)=\frac{P\left(D|M_{n}\right)P\left(M_{n}|M\right)}{\sum_{n=1}^{N}P\left(D|M_{n}\right)P\left(M_{n}|M\right)}$$

Here, $M\equiv \left\{M_{n}:n=1,2,\ldots ,N\right\}$ where $M_{n}$ represents the model class corresponding to a particular nth grouting condition, and N is the total number of grouting conditions considered, including partial grouting and cavity defects. D denotes the set of measurements. $P\left(M_{n}|M\right)$ is the prior probability of the $M_{n}$ model class, and $\sum_{n=1}^{N}P\left(M_{n}|M\right)=1$. The value of the prior possibility of each model class is set to its frequency, which equals 1/N in this study. Actually, the influence of the prior possibility value on the identification precision would become smaller when the sample number increases big enough.

It can be seen that the evaluation of the post possibility of $M_{n}$ from the measurement data, namely $P\left(M_{n}|D,M\right)$, requires the distribution of $P\left(D|M_{n}\right)$. In this study, the energy of WPT components was assumed and also testified using experimental measurements to follow the normal distribution. As for comparison, the energy vector of eight WPT components and the total energy index were respectively applied as the input of Bayes classifier. For the energy vector of eight WPT components, the probability density function can be represented as

(8) $$P\left(D|M_{n}\right)={\left(2\pi \right)}^{-\frac{2^{j}}{2}}{\left|S_{n}\right|}^{-\frac{1}{2}}\exp\left[-\frac{1}{2}{\left(D-\bar{D_{n}}\right)}^{T}{S_{n}}^{-1}\left(D-\bar{D_{n}}\right)\right]$$

where $D=\left(E_{1},E_{2},\ldots ,E_{2^{j}}\right)$ is the $2^{j}$-dimensional random vector composed by the energies of WPT components. $S_{n}$ is the $2^{j}$-dimensional covariance matrix, which is calculated by $S_{n}=E\left[\left(D-\bar{D_{n}}\right){\left(D-\bar{D_{n}}\right)}^{T}\right]$. $\bar{D_{n}}$ is the mean value of nth grouting condition.

The classification of one certain testing data can be realized by calculating and comparing the possibility values of Equation (7) for all the model classes. The most appropriate class corresponds to the highest possibility. By taking the energy vector of WPT components as the input, the Bayes classifier makes full use of the time-frequency characteristics of received signals and contributes to more accurate classification of grouting conditions in post-tensioning tendon ducts.

## 3. Experimental Procedure

### 3.1. PZT Transducers

**Figure 3:**
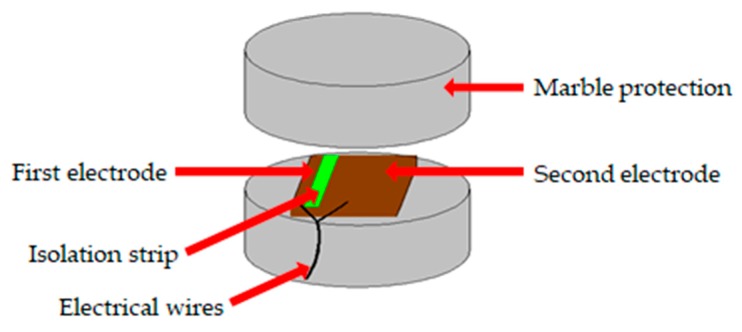
Schematic of marble-encased lead zirconate titanate (PZT) patch used as the actuator.

Piezoelectric ceramic is able to convert electrical and mechanical energy into each other. This property is called piezoelectricity. Piezoelectric material will generate electric charge when subjected to strain, namely the positive (direct) effect. In the converse piezoelectric effect, piezoelectric material will produce strain when it is polarized by electric field. Piezoelectric materials can thus be used as both actuators and sensors because of the positive and converse piezoelectric effect. In this study, PZT transducers were used to generate and receive ultrasonic stress waves. One PZT patch sandwiched with marble protection (Figure 3), namely SA, was bonded on the prestressing reinforcement and used as the actuator to generate stress waves. Four PZT patches were mounted on the tendon duct surface and utilized as the sensors to receive stress waves over a certain distance.

### 3.2. Specimens with Different Grouting Conditions

**Figure 4:**
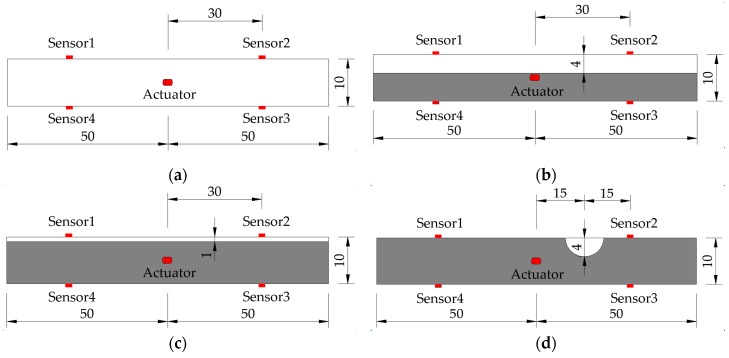

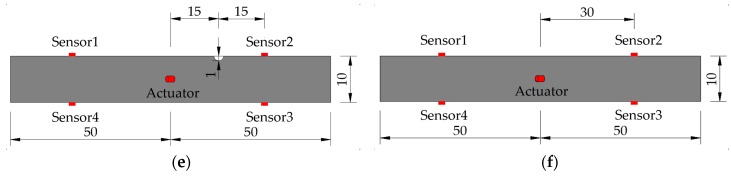
Design of tendon duct specimens with various grouting defects (unit: cm): (**a**) 0%-grouting; (**b**) 60%-grouting; (**c**) 90%-grouting (**d**) 4 cm-cavity; (**e**) 1 cm-cavity; (**f**) 100%-grouting.

**Figure 5:**
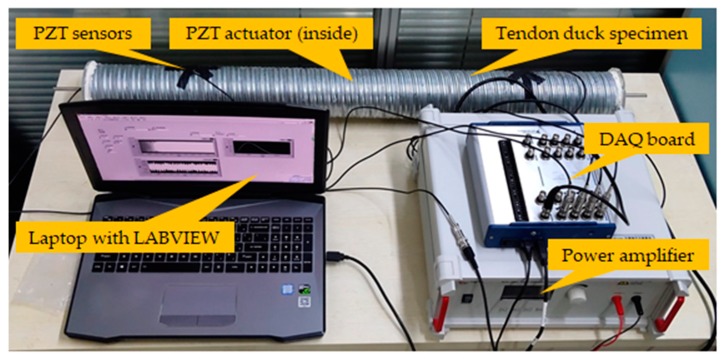
Experimental setup.

There are various kinds of tendon duct grouting defects in practice. Bleeding of the grouting mortar introduces cavities in the upper part of the transverse tendon duct. Grouting mortar leakage and no pressure stabilizing step in the end of the grouting process would cause diverse partial grouting conditions. Steel wires used to tie the prestressing steel strands together may accumulate at the deformed or curved sections when the prestressing tendon passed through the duct. There may be other sundries, such as waste concrete, stacked inside the duct because of nonstandard operations in practice. Moreover, the vent pipe may be blocked during the concrete casting. These factors would block the mortar in the grouting process and further result in the 0%-grouting condition. In this context, six specimens were designed, as shown in Figure 4, respectively, with three partial grouting defects (0%-grouting, 60%-grouting, and 90%-grouting), two cavities defects (4 cm-diameter spherical cavity and 1 cm-diameter spherical cavity), and one 100%-grouting as the benchmark. The specimens were fabricated to reproduce the typical grouting defects in practice as much as possible. The length of tendon duct specimens was 100 cm, the diameter was 10 cm, and the thickness was 1 mm. Acrylic plastic plates were used to seal the ends of tendon ducts. The specimens were cured for ten days before testing. The experimental setup is shown in Figure 5.

### 3.3. Data Acquisition

**Figure 6:**
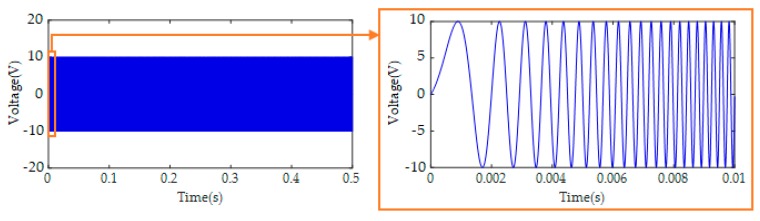
The swept sine signal used to motivate PZT actuator.

As shown in Figure 5, the data acquisition system included PZT transducers, a NI-USB-6366 data acquisition (DAQ) board (supplied by National Instruments Corp., Austin, TX, USA), a power amplifier, and a laptop installed with the LABVIEW software. Swept sine signals were generated by the LABVIEW software and transformed to voltage signals by the DAQ board. The amplitude and period of the swept sine signals (Figure 6) were 10 V and 0.5 s, respectively. The frequency range was from 100 Hz to 200 kHz, as listed in Table 1. The sampling frequency of data acquisition was 2 MHz. The power amplifier helped to amplify the swept sine voltage signals by 50 times so that the voltage was high enough to motivate the PZT actuator and generate ultrasonic stress waves with expected amplitudes. The data acquisition board did not only generate swept sine signals for the PZT actuator but also received stress wave signals from the PZT sensors. The LABVIEW software controlled the whole data acquisition procedure and stored the data in the laptop. Each specimen with a specific grouting condition was tested 60 times in order to obtain enough data with randomness in this study. In each test, a swept sine signal of 0.5 s was generated by the data acquisition board and input to the specimen through the PZT actuator. Simultaneously, a response of 0.5 s was collected by the PZT sensors and the data acquisition board.

## 4. Results and Discussion

### 4.1. Waveforms and WPTs of Different Grouting Conditions

**Figure 7:**
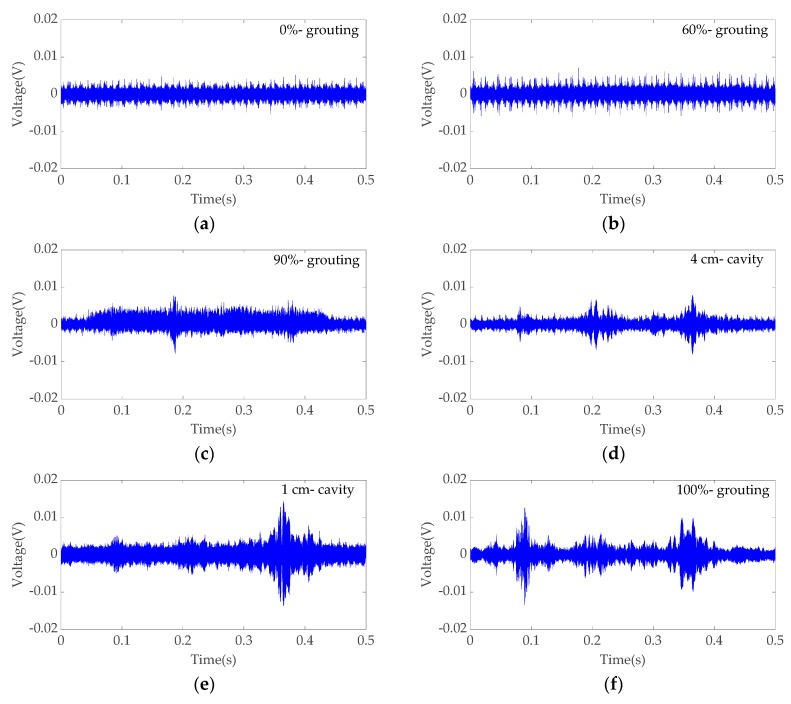
Example signals received by Sensor 2 in six grouting conditions: (**a**) 0%-grouting; (**b**) 60%-grouting; (**c**) 90%-grouting; (**d**) 4 cm-cavity; (**e**) 1 cm-cavity; (**f**) 100%-grouting.

The received stress wave signals have certain randomness in practice. Each specimen of a specific grouting condition was tested 60 times in order to obtain enough data with randomness. The stress wave signals received by Sensor 2 were analyzed here. Figure 7 shows six typical signals, of which the amplitudes and energies are close to the average values of 60 readings for the six grouting conditions. As can be seen, the maximum amplitudes of signals for 0%-grouting and 60%-grouting conditions were, respectively, 0.0054 V and 0.0071 V, which were in similar level; the maximum amplitudes of signals for 90%-grouting and 4 cm-cavity conditions were, respectively, 0.0079 V and 0.0081 V, which were in very similar level; and the maximum amplitudes of signals for 1 cm-cavity and 100%-grouting conditions were, respectively, 0.0145 V and 0.0133 V, which were in very similar level. Even more, the maximum amplitudes of signals for 1 cm-cavity and 100%-grouting conditions were not consistent with the grouting compactness. Therefore, it was difficult to distinguish different grouting conditions using the maximum amplitude of signal in the time domain.

**Figure 8:**
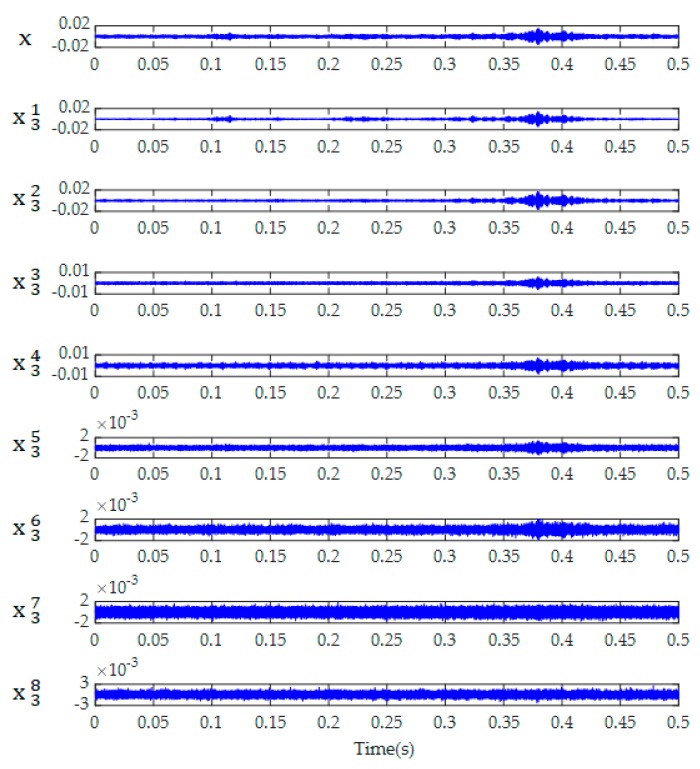
WPT components of a signal received in the grouting condition with 1 cm-cavity.

**Figure 9:**
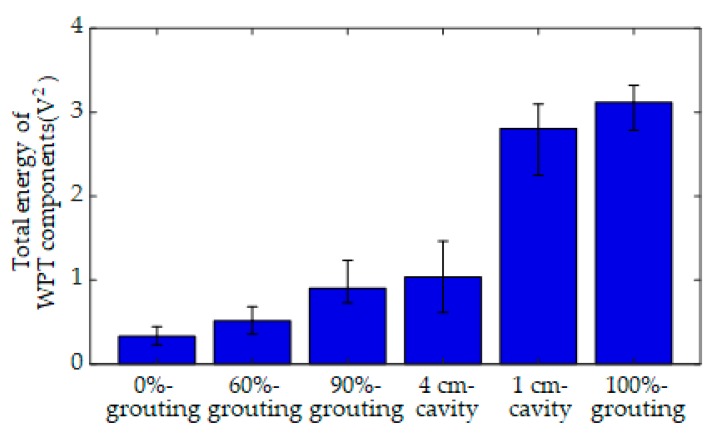
The total energy of WPT components for six grouting conditions.

The three-level WPT was therefore applied to analyze the received signals and to decompose them into eight frequency scales. As an example, Figure 8 shows the eight reconstructed WPT components of a signal received in the grouting condition with 1 cm-cavity, which is given in Figure 7e. After collecting 60 data samples for each specimen, the total energy of WPT components were calculated for each data sample. The mean value and variation range of the total energy of 60 data samples were obtained for each grouting condition, as shown in Figure 9 and Table 2. The height of the bars represents the mean value of the total energy, and the error bar represents the variation range of the total energy for different grouting conditions. It can be seen that the mean value of the total energy increased along with the increase of grouting compactness. However, the variation ranges of the total energy for different grouting conditions seriously overlapped with each other due to the randomness of practical measurements. For example, the mean values of the total energy for 90%-grouting and 4 cm-cavity were, respectively, 0.903 and 1.032, which were very close; however, the variation ranges of the total energy for these two grouting conditions were, respectively, [0.728–1.234] and [0.606–1.430], which were seriously overlapped. That is, the total energy of 90%-grouting in average was slightly lower than that of 4 cm-cavity; however, the total energy of 90%-grouting in one test could be larger than that of 4 cm-cavity. It easily led to misidentification between these two grouting conditions. Similarly, the variation ranges of the total energy for 0-grouting, 60%-grouting, and even 4 cm-cavity were overlapped, and those of the total energy for 1 cm-cavity and 100%-grouting were also overlapped. It was therefore not appropriate to utilize the total energy solely as the classification criterion for grouting quality evaluation.

**Figure 10:**
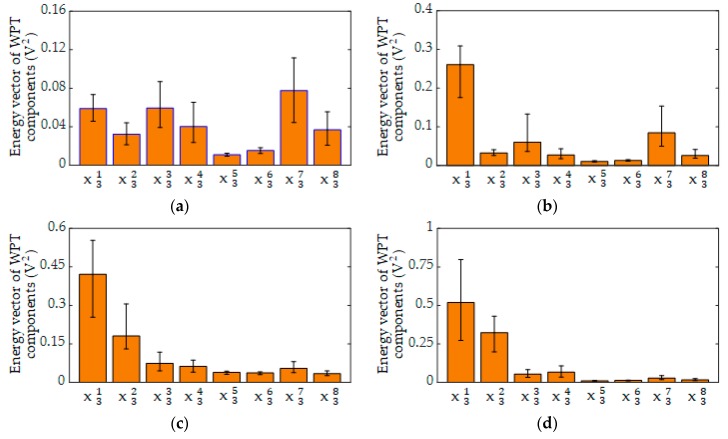

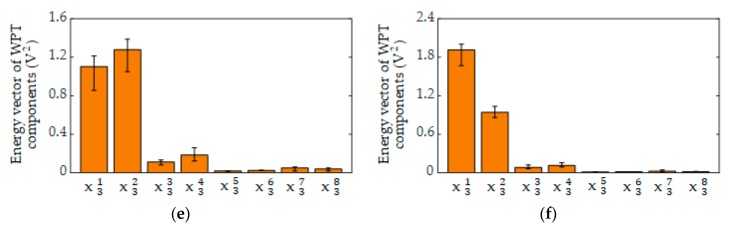
The energy vector of WPT components for six grouting conditions: (**a**) 0% grouting; (**b**) 60%-grouting; (**c**) 90%-grouting (**d**) 4 cm-cavity; (e) 1 cm-cavity; (**f**) 100%-grouting.

Then, the respective energies of all the WPT components were calculated for all the signals received in the six grouting conditions. The mean value and variation range of the energy vector of WPT components for each grouting condition are shown in Figure 10 and Table 2. There were still overlaps in the energy values of some WPT components, such as the variation range of the first WPT component for 90-grouting ([0.728–1.234]) and that for 4-cavity ([0.606–1.430]). However, it was obvious that the energy distribution of the received signals in the eight frequency scales varied among the six grouting conditions. For example, the average energy vectors of WPT components for 90%-grouting and 1 cm-cavity were, respectively, [0.903, 0.421, 0.181, 0.074, 0.063, 0.039, 0.037, 0.054, 0.034] and [1.032, 0.519, 0.324, 0.054, 0.067, 0.011, 0.013, 0.028, 0.017], which are different with each other. These patterns of the energy distribution could then be used to distinguish different grouting conditions more accurately.

### 4.2. Grouting Quality Evaluation Using Bayes Classifier

**Figure 11:**
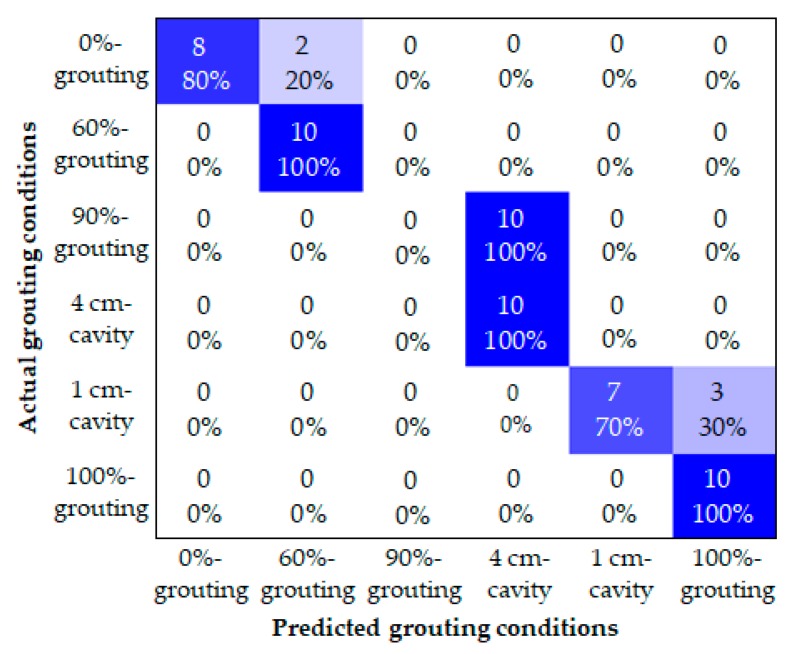
Confusion matrix of the Bayes classifier with the total energy as input.

**Figure 12:**
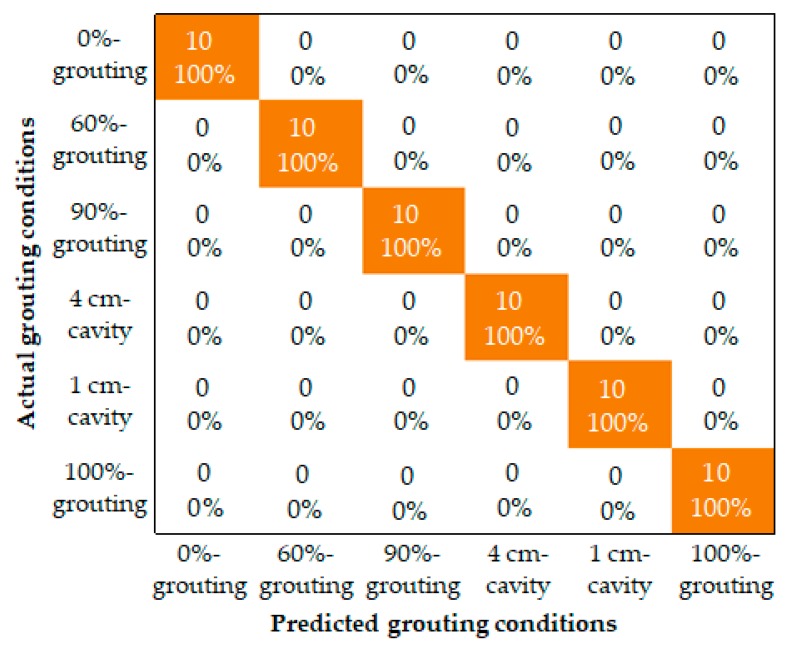
Confusion matrix of the Bayes classifier with the energy vector as input.

As for comparison, the Bayes classifier method was applied to identify different grouting conditions by, respectively, taking the total energy and the energy vector of WPT components as the input. Since each tendon duct specimen was tested for 60 times, there are in total 360 data samples for all the grouting conditions. Three hundred data samples were used to independently train the two Bayes classifiers with different inputs, and 60 data samples were used to test and compare their performances. The overall classification accuracies of the two Bayes classifiers input with the total energy and the energy vector were, respectively, 75% and 100%. The confusion matrixes of corresponding classification results are respectively displayed in Figure 11 and Figure 12. With the total energy as input, the Bayes classifier mistakenly classified 20% of the 0%-grouting conditions to 60%-grouting conditions, classified all the 90%-grouting conditions to 4 cm-cavity conditions, and classified 30% of the 1 cm-cavity conditions to 100%-grouting conditions. On the other hand, with the energy vector of eight WPT components as input, the Bayes classifier achieved 100% classification accuracy for all the grouting conditions. The energy vector of WPT components was proved to perform better than the traditionally used total energy in the classification of different grouting conditions.

### 4.3. Results Discussion

The proposed method based on the Bayes classifier input with the energy vector of WPT components of received stress waves successfully identified the six grouting conditions investigated with 100% classification accuracy. WPT decomposed the analyzed signals both in high-frequency regions and low-frequency regions, and helped to clearly explore the energy distribution of stress waves in multi-frequency scales. The frequency components of stress waves had various sensitivities to different grouting conditions. The energy distribution of WPT components could therefore classify different grouting conditions more precisely than the total energy of WPT components that equaled the total energy of stress waves. It was evident that the energy vector provided more detailed time-frequency characteristics of received signals for classification, and that the Bayes classifier further helped to make an accurate grouting quantity evaluation.

Although more grouting conditions have been investigated here than previous studies, it is impossible to cover all the possible grouting scenarios in practice for training this classification method. In fact, it is a problem for most defect diagnosis methods based on data clustering or classification. If an unexpected grouting scenario occurs, the identification result of the proposed method would be the most similar class investigated in this study. For examples, a 50%-grouting condition would be identified to be the 60%-grouting condition, and a 2 cm-cavity condition would be identified to be the 1 cm-cavity condition. In the future, more typical grouting conditions can be involved in the proposed method to improve its applicability and effectiveness in practice.

## 5. Conclusions

In this study, a method based on WPT and Bayes classifier was proposed to evaluate grouting quality in post-tensioning tendon ducts by using stress waves generated and received by piezoelectric transducers. One PZT transducer bonded on the prestressing reinforcement was used as the actuator, while four PZT transducers mounted on the surface of the duct were utilized as the sensors. Six typical grouting conditions with both partial grouting and cavity defects of different dimensions were experimentally investigated. Due to the randomness of practical measurements, the variation ranges of the energy of stress waves received in different grouting conditions overlapped to various degrees. The WPT was thus applied to explore the energy distribution of received stress wave signals at multi-scales. After that, the Bayes classifier was employed to take into account the randomness of practical measurements in the grouting quality evaluation. As for comparison, the traditionally used total energy and the proposed energy vector of WPT components were, respectively, input to the Bayes classifier. The overall classification accuracy of the Bayes classifier input with the total energy was 75%, while that of the Bayes classifier input with the energy vector achieved 100%. The experimental results demonstrated that the Bayes classifier input with the energy vector overcame the shortcoming of using only the total energy as the classification criterion and could identify grouting conditions more accurately. The proposed grouting quality evaluation method has the potential to be applied at key spots of post-tensioning tendon ducts in practice.

For future work, more grouting conditions especially the cavity at different positions will be studied through finite element simulation and experiments. The effects of water existing in grouting defects and post-tensioning tendons on the grouting quality evaluate would be figured out. The propagation path of stress waves in various grouting conditions will be investigated in order to involve more typical grouting conditions in the classification study.

## Figures and Tables

**Table 1:** Parameters of the swept sine signal.

Start Frequency	Stop Frequency	Amplitude	Period
100 Hz	200 kHz	10 V	0.5 s

**Table 2:** Mean values and variation ranges of the total energy and the energy vector of WPT components for six grouting conditions.

Grouting Conditions		Total	$x_{3}^{1}$	$x_{3}^{2}$	$x_{3}^{3}$	$x_{3}^{4}$	$x_{3}^{5}$	$x_{3}^{6}$	$x_{3}^{7}$	$x_{3}^{8}$
0%-grouting	Mean	0.329	0.059	0.032	0.059	0.040	0.011	0.015	0.078	0.037
	Varia-tion	[0.225–0.445]	[0.045–0.073]	[0.021–0.044]	[0.039–0.086]	[0.023–0.065]	[0.009–0.012]	[0.012–0.018]	[0.044–0.111]	[0.020–0.055]
60%-grouting	Mean	0.516	0.261	0.033	0.061	0.027	0.011	0.013	0.085	0.026
	Varia-tion	[0.357–0.681]	[0.175–0.308]	[0.025–0.041]	[0.036–0.133]	[0.017–0.043]	[0.009–0.012]	[0.011–0.015]	[0.049–0.153]	[0.018–0.041]
90%-grouting	Mean	0.903	0.421	0.181	0.074	0.063	0.039	0.037	0.054	0.034
	Varia-tion	[0.728–1.234]	[0.254–0.553]	[0.130–0.305]	[0.045–0.118]	[0.039–0.087]	[0.031–0.043]	[0.030–0.041]	[0.037–0.081]	[0.026–0.044]
4 cm-cavity	Mean	1.032	0.519	0.324	0.054	0.067	0.011	0.013	0.028	0.017
	Variation	[0.606–1.430]	[0.273–0.798]	[0.198–0.430]	[0.032–0.082]	[0.035–0.108]	[0.009–0.011]	[0.010–0.014]	[0.018–0.044]	[0.012–0.025]
1 cm-cavity	Mean	2.807	1.102	1.278	0.112	0.184	0.017	0.024	0.051	0.039
	Variation	[2.251–3.096]	[0.855–1.213]	[1.049–1.387]	[0.082–0.132]	[0.121–0.259]	[0.015–0.018]	[0.020–0.027]	[0.018–0.060]	[0.018–0.050]
100%-grouting	Mean	3.119	1.915	0.943	0.082	0.112	0.012	0.016	0.024	0.014
	Variation	[2.787–3.321]	[1.667–2.005]	[0.861–1.035]	[0.063–0.121]	[0.088–0.154]	[0.011–0.013]	[0.014–0.017]	[0.014–0.045]	[0.010–0.021]
